# Supplementary material for: Splicing Machinery is Dysregulated in Pituitary Neuroendocrine Tumors and is Associated with Aggressiveness Features
Source: Cancers (Basel). 2019 Sep 26;11(10):1439. doi: 10.3390/cancers11101439 (PMC6826715; doi:10.3390/cancers11101439)
Supplement: Supplementary file 1 [file cancers-11-01439-s001.pdf]

Supplemental Material

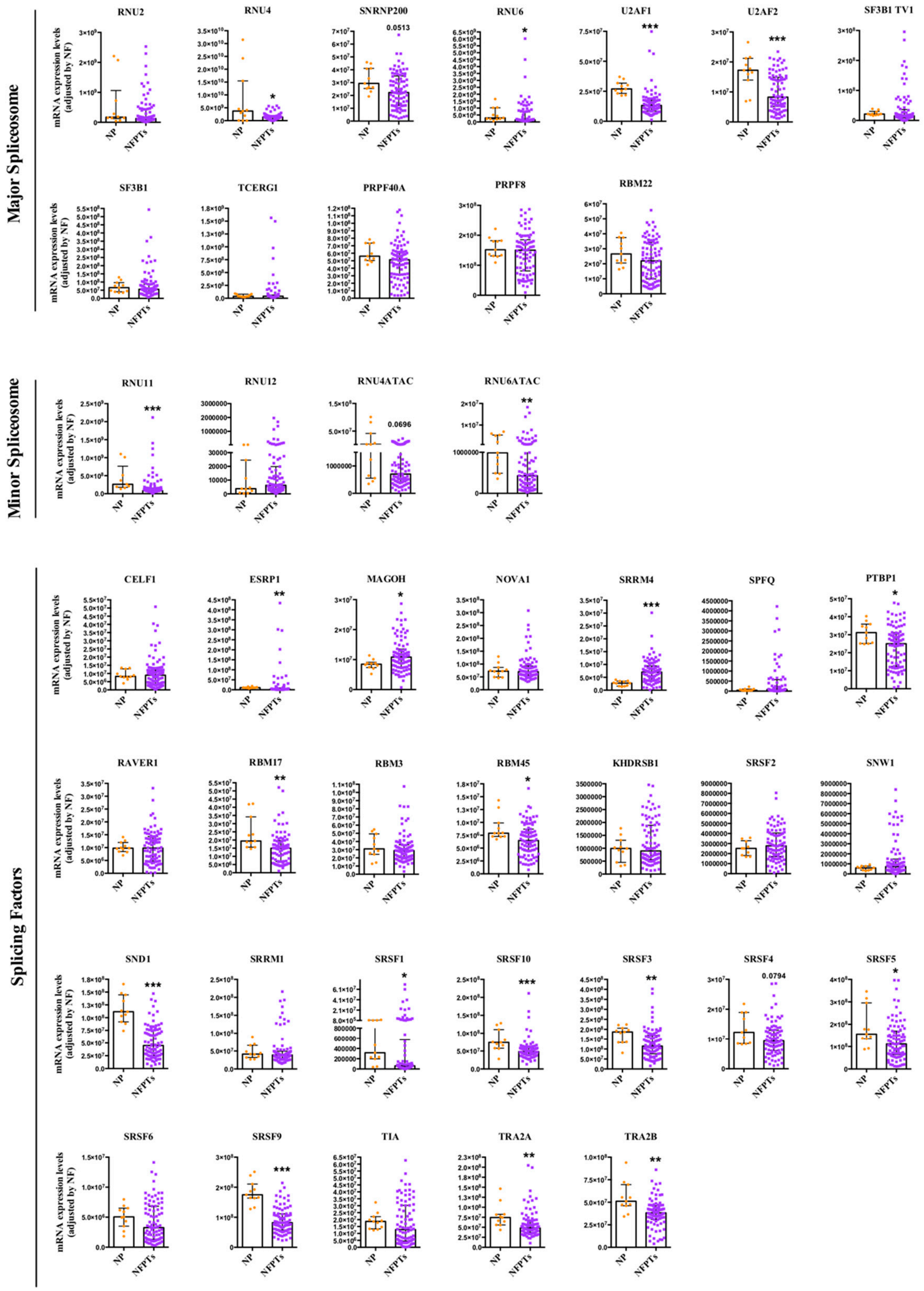

Figure S1. Non-functioning pituitary tumors (NFPTs). mRNA expression levels of all spliceosome components and splicing factors measured in NFPTs compared to NPs using the qPCR array. Data represent median ± interquartile range of absolute expression levels (copy number) of each transcript adjusted by a normalization factor. Asterisks (* $p < 0.05$, ** $p < 0.01$, *** $p < 0.001$) indicate statistically significant differences between groups.

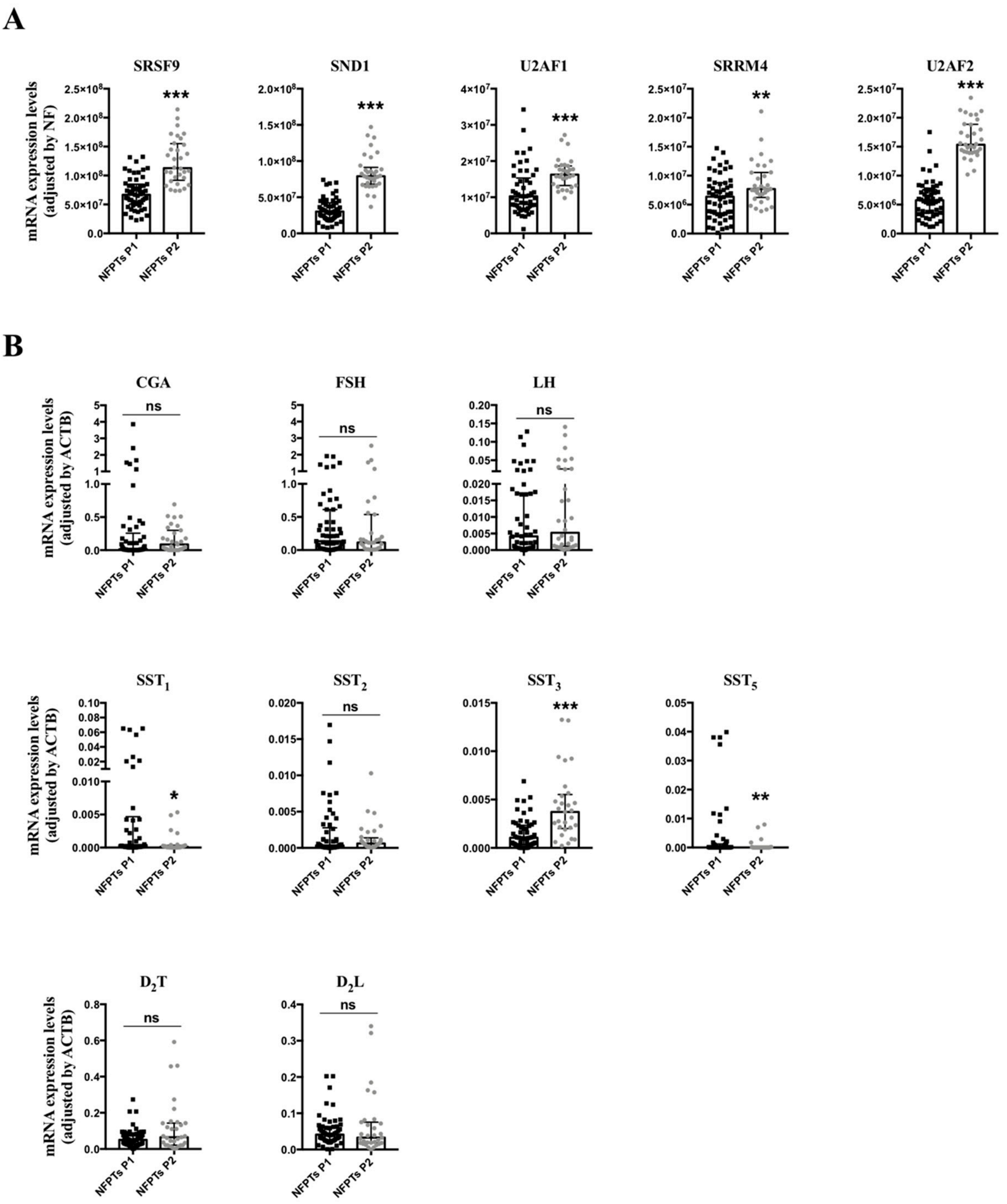

Figure S2. Non-functioning pituitary tumors (NFPTs). (**A**) mRNA expression levels of SRSF9, SND1, U2AF1, SRRM4 and U2AF2 in the two population of NFPTs (P1 and P2) observed in the heatmap. Data represent median ± interquartile range of absolute expression levels (copy number) of each transcript measured by qPCR and adjusted by a normalization factor. (**B**) mRNA expression levels of classical hormones (CGA, FSH and LH) and somatostatin and dopamine receptors ($SST_{1–5}$ and D_2) in the two population of NFPTs observed in the heatmap. Data represent median ± interquartile range of absolute expression levels (copy number) of each transcript measured by qPCR and adjusted by β-actin (ACTB). Asterisks (* $p < 0.05$, ** $p < 0.01$, *** $p < 0.001$) indicate statistically significant differences between groups.

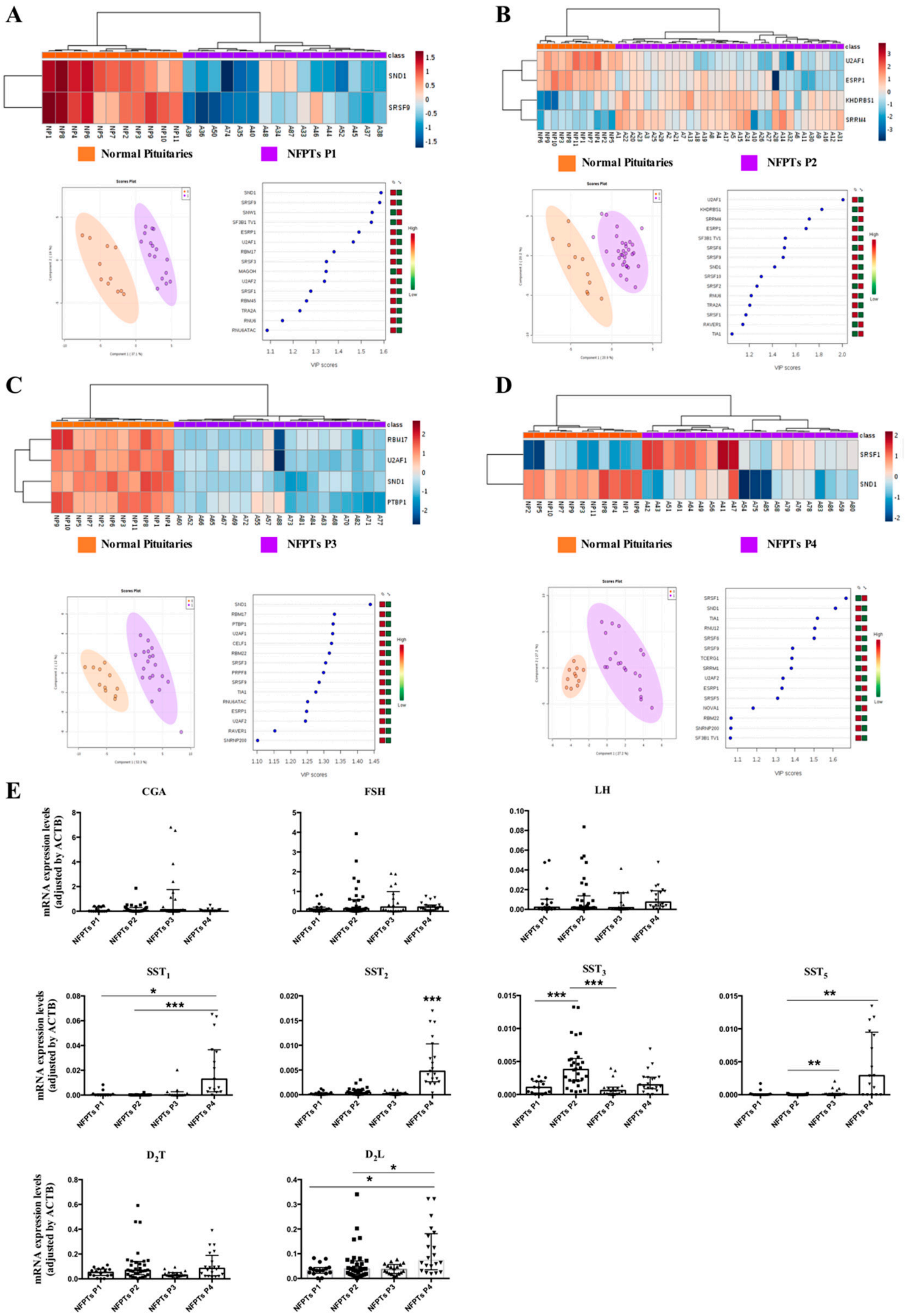

Figure S3. Non-functioning pituitary tumors (NFPTs). (**A–D**) Heatmaps of the splicing-regulatory elements with higher VIP score in the different populations of NFPTs (P1–P4) analyzed. Principal Components Analysis (PCA) of the mRNA expression levels of all the splicing-regulatory elements analyzed and VIP Scores top-feature of Partial Least Squares Discriminant Analysis (PLS-DA). (**E**) mRNA expression levels of classical hormones (CGA, FSH and LH) and somatostatin and dopamine receptors (SST_{1-5} and D_2) in the four populations of NFPTs observed in the first heatmap. Data represent median ± interquartile range of absolute expression levels (copy number) of each transcript measured by qPCR and adjusted by β-actin (ACTB). Asterisks (* $p < 0.05$, ** $p < 0.01$, *** $p < 0.001$) indicate statistically significant differences between groups.

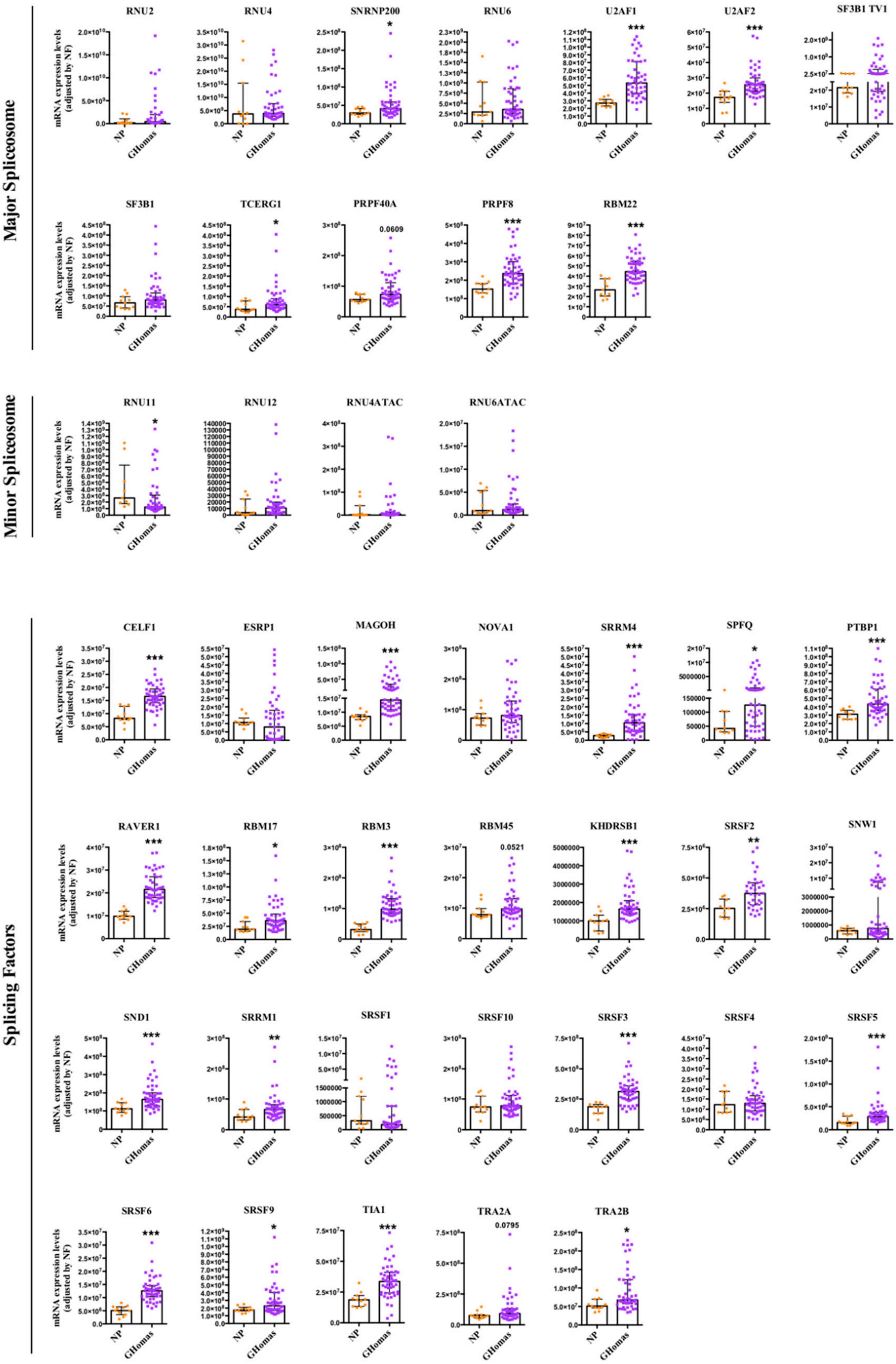

Figure S4. Somatotropinomas (GHomas). mRNA expression levels of all spliceosome components and splicing factors measured in GHomas compared to NPs using the qPCR array. Data represent median ± interquartile range of absolute expression levels (copy number) of each transcript adjusted by a normalization factor. Asterisks (* $p < 0.05$, ** $p < 0.01$, *** $p < 0.001$) indicate statistically significant differences between groups.

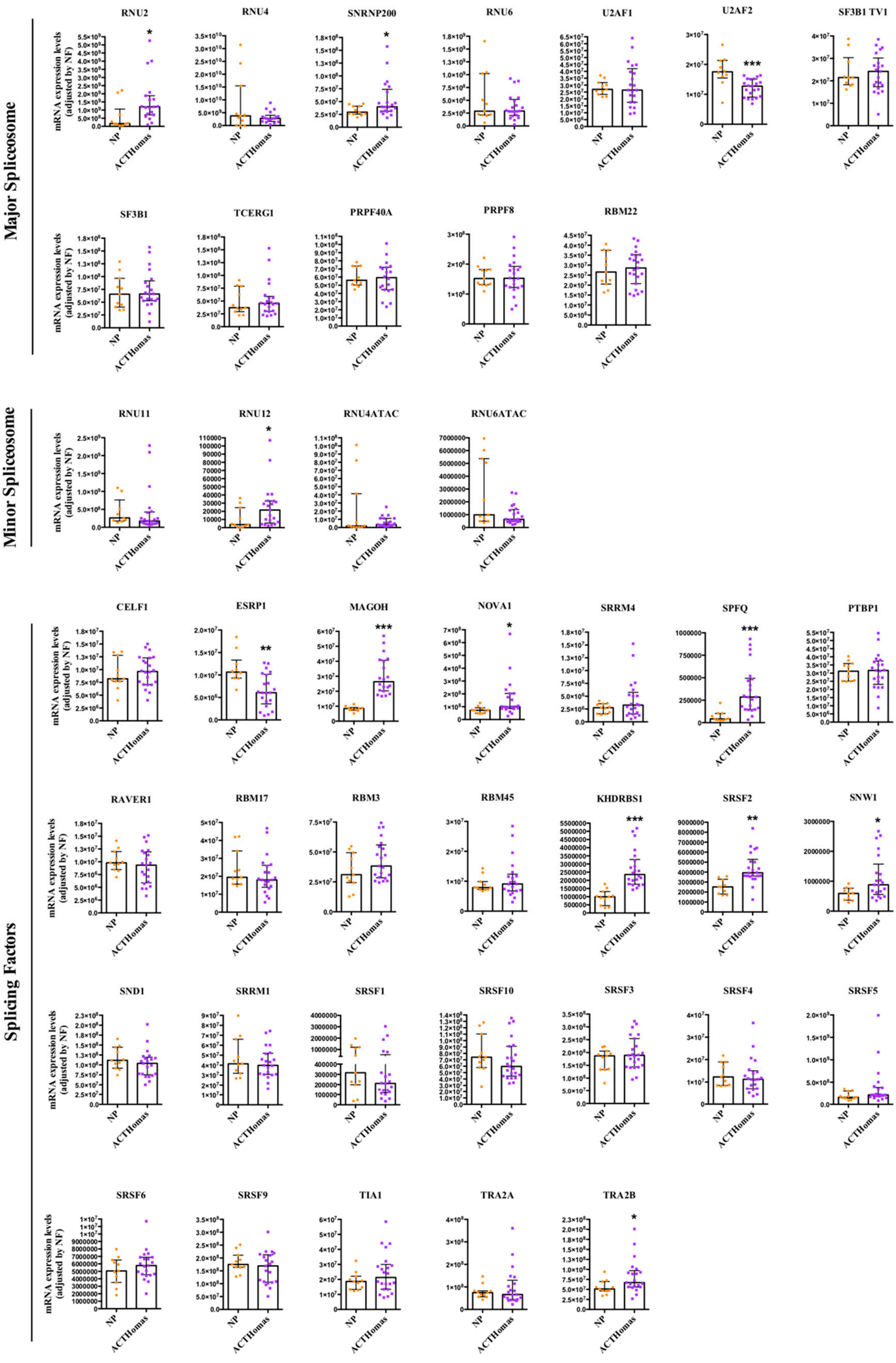

Figure S5. Corticotropinomas (ACTHomas). mRNA expression levels of all spliceosome components and splicing factors measured in ACTHomas compared to NPs using the qPCR array. Data represent median ± interquartile range of absolute expression levels (copy number) of each transcript adjusted by a normalization factor. Asterisks (* $p < 0.05$, ** $p < 0.01$, *** $p < 0.001$) indicate statistically significant differences between groups.

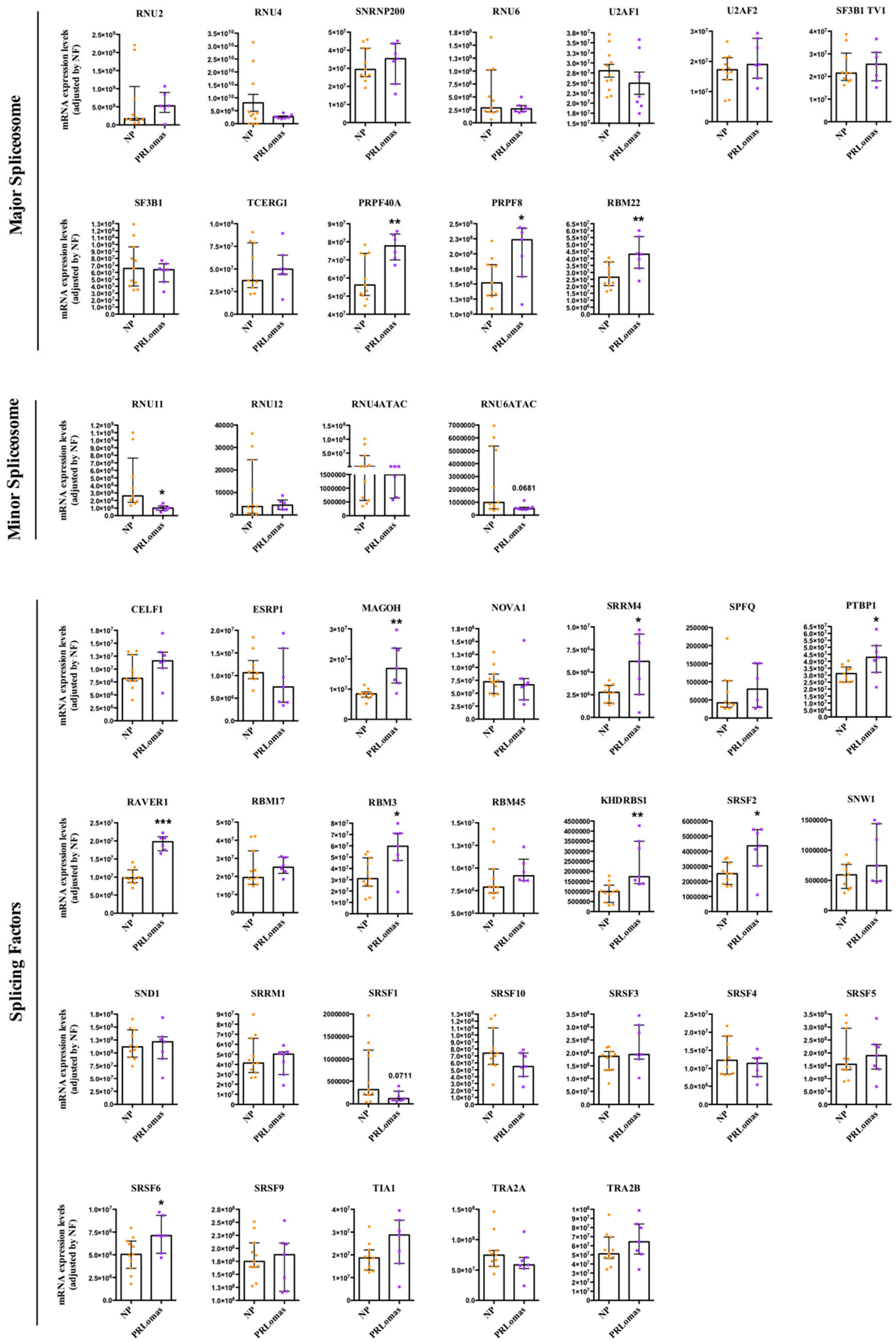

Figure S6. Prolactinomas (PRLomas). mRNA expression levels of all spliceosome components and splicing factors measured in PRLomas compared to NPs using the qPCR array. Data represent median ± interquartile range of absolute expression levels (copy number) of each transcript adjusted by a normalization factor. Asterisks (* $p < 0.05$, ** $p < 0.01$, *** $p < 0.001$) indicate statistically significant differences between groups.

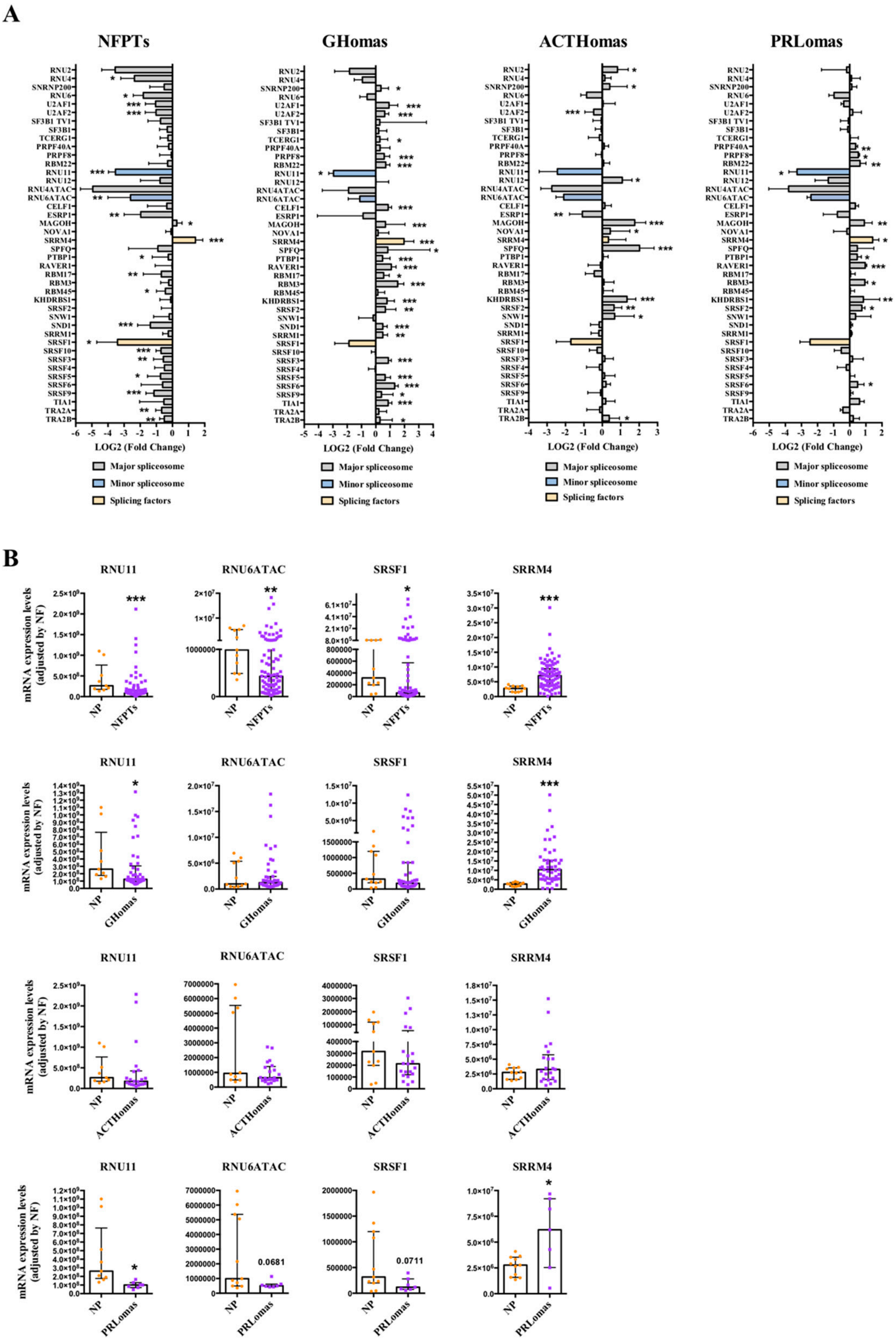

Figure S7. (**A**) Individual Fold-Change of spliceosome machinery expression levels showing the common dysregulated components (blue and yellow colors) in all PitNETs subtypes compared to NPs. (**B**) mRNA expression levels of spliceosome components and the splicing factor commonly dysregulated in NFPTs, GHomas, ACTHomas, and PRLomas, respectively. Data represent median ± interquartile range of absolute expression levels (copy number) of each transcript adjusted by a normalization factor. Asterisks (* $p < 0.05$, ** $p < 0.01$, *** $p < 0.001$) indicate statistically significant differences between groups.

Table S1. Results from Chi-square test of clinical parameters between P1 and P2 of NFPTs derived from the second heatmap.

Clinical Parameters	Chi-Squared Test	*p*-value
Sex	0.141	0.707
Cephalea	2.462	0.292
Visual alterations	0.201	0.904
Extrasellar growth	1.377	0.241
Cavernous sinus invasion	0.575	0.448
Chiasmatic compression	0.967	0.325
Pre-surgery treatment	1.469	0.225
Ki67 (<2% or ≥2%)	0.650	0.420
Curation	0.001	0.980

Table S2. Results from Chi-square test of clinical parameters between P1, P2, P3 and P4 of NFPTs derived from the first heatmap (cutting the dendogram at second highest height).

Clinical Parameters	Chi-Squared Test	*p*-value
Sex	2.344	0.504
Cephalea	8.461	0.206
Visual alterations	3.722	0.714
Extrasellar growth	1.123	0.772
Cavernous sinus invasion	2.859	0.414
Chiasmatic compression	0.240	0.971
Pre-surgery treatment	4.104	0.250
Ki67 (<2% or ≥2%)	0.436	0.933
Curation	1.796	0.616
